# Structural Characteristics of Simple RNA Repeats Associated with Disease and their Deleterious Protein Interactions

**DOI:** 10.3389/fncel.2017.00097

**Published:** 2017-04-11

**Authors:** Adam Ciesiolka, Magdalena Jazurek, Karolina Drazkowska, Wlodzimierz J. Krzyzosiak

**Affiliations:** Department of Molecular Biomedicine, Institute of Bioorganic Chemistry, Polish Academy of SciencesPoznan, Poland

**Keywords:** simple expanded repeats, repeat expansion disorders, RNA repeat structures, RNA toxicity, RNA-binding proteins

## Abstract

Short Tandem Repeats (STRs) are frequent entities in many transcripts, however, in some cases, pathological events occur when a critical repeat length is reached. This phenomenon is observed in various neurological disorders, such as myotonic dystrophy type 1 (DM1), fragile X-associated tremor/ataxia syndrome, C9orf72-related amyotrophic lateral sclerosis and frontotemporal dementia (C9ALS/FTD), and polyglutamine diseases, such as Huntington’s disease (HD) and spinocerebellar ataxias (SCA). The pathological effects of these repeats are triggered by mutant RNA transcripts and/or encoded mutant proteins, which depend on the localization of the expanded repeats in non-coding or coding regions. A growing body of recent evidence revealed that the RNA structures formed by these mutant RNA repeat tracts exhibit toxic effects on cells. Therefore, in this review article, we present existing knowledge on the structural aspects of different RNA repeat tracts as revealed mainly using well-established biochemical and biophysical methods. Furthermore, in several cases, it was shown that these expanded RNA structures are potent traps for a variety of RNA-binding proteins and that the sequestration of these proteins from their normal intracellular environment causes alternative splicing aberration, inhibition of nuclear transport and export, or alteration of a microRNA biogenesis pathway. Therefore, in this review article, we also present the most studied examples of abnormal interactions that occur between mutant RNAs and their associated proteins.

## Introduction

In the human genome, Short Tandem Repeats (STRs, typically 1–6 nucleotide repeats) are common in intergenic regions and in untranslated and translated regions of protein-coding genes. Such repeats are characterized by genetic instability as well as by an ability to expand (Ellegren, 2004). Mutational expansions of certain types of STRs occurring in either coding or non-coding regions of functionally unrelated genes are causative factors for more than 20 inherited human genetic disorders involving the nervous system. These typically late-onset diseases include myotonic dystrophy type 1 (DM1) and 2 (DM2), fragile X syndrome (FXS), fragile X-associated tremor ataxia syndrome (FXTAS), *C9orf72* amyotrophic lateral sclerosis and frontotemporal dementia (C9ALS/FTD), Friedreich’s ataxia (FRDA) and nine polyglutamine diseases, such as Huntington’s disease (HD) and a number of spinocerebellar ataxias (SCA). DM1 and DM2 are caused by a CTG expansion (50 to >3500) in the 3′UTR of the dystrophia myotonica protein kinase (*DMPK*) gene and by a CCTG expansion (75 to approximately 11000) in the first intron of the zinc finger protein 9 (*ZNF9*) gene, respectively (Ranum and Cooper, 2006; O’Rourke and Swanson, 2009; Thornton, 2014). FXTAS is triggered by CGG tracts in the 5′UTR of the fragile X mental retardation 1 (*FMR1*) gene (55–200 repeats; Hagerman, 2013; Hagerman and Hagerman, 2013). When the CGG expansion exceeds more than 200 repeats in the same gene, FXS occurs. Hundreds to thousands of GGGGCC repeats in the first intron of the chromosome 9 open reading frame 72 (*C9orf72*) gene represent the most common genetic abnormality in C9ALS/FTD (DeJesus-Hernandez et al., 2011; Renton et al., 2011; Smith et al., 2013). The exact pathogenic size of GGGGCC is not well established; however, the presence of less than 30 repeats is generally not associated with disease. The abnormal expansion of GAA repeats (66 to more than 1000) located in the first intron of the frataxin (*FXN*) gene is the causative agent of FRDA (Orr and Zoghbi, 2007). In the most common polyQ disorders, HD and SCA3, the expression of at least 36 CAG repeats in the first exon of the huntingtin (*HTT*) gene and of at least 60 CAG repeats in the 10th exon of the ataxin 3 (*ATXN3*) gene, respectively, is sufficient to cause pathogenic effects (Orr and Zoghbi, 2007).

Depending on the location within genes, three primary mechanisms by which simple repeat expansion could contribute to pathogenesis have been distinguished: (1) a toxic RNA gain-of-function mechanism in which expanded toxic RNA species tend to form intracellular RNA foci that sequester important proteins from their normal cellular functions; (2) a toxic protein gain-of-function mechanism in which the presence of polyQ stretches encoded by elongated CAG repeats results in protein conformational changes, altered protein-protein interactions and aggregate formation; and (3) aberrant loss-of-transcript and loss-of-protein functions in which transcript or protein expression is inhibited by the expanded repeats. However, taking into account the occurrence of bidirectional transcription across the expanded repeats (Moseley et al., 2006; Ikeda et al., 2008; Batra et al., 2010) as well as the more recently discovered repeat-associated non-AUG (RAN) translation (Zu et al., 2011; Ash et al., 2013; Mori et al., 2013; Todd et al., 2013; Wojciechowska et al., 2014), the pathogenic complexity of repeat expansion disorders, particularly those caused by non-coding sequences, further increases.

For over a decade, intensive studies have been undertaken to determine how mutant RNAs containing long repeat tracts might trigger neurodegeneration. In particular, the structure of repeat RNAs is under investigation, as it is strongly believed that it functions as a causative agent. As the RNA-dominant mechanism is strictly associated with the sequestration of diverse proteins by nuclear aggregates that are formed by expanded repeats, many efforts have also been undertaken to identify mutant RNA-binding proteins. In this review article, we present detailed information regarding the RNA structure of disease-relevant simple repeats. We also briefly describe examples of the most-studied interactions between repeat RNAs and their interacting proteins.

## Biochemical and Biophysical Studies on Simple RNA Sequence Repeats

Many methods have been applied to analyze RNA repeat structures. Using *in vitro* biochemical and biophysical analyses, it has been revealed that repeat RNAs can adopt diverse secondary structures from semistable hairpins to fairly stable hairpins by very stable quadruplexes, depending on the type of expanded motif. As presented below, in most studies investigating mutant RNA structures, pure tandem repeats were used. Only a few reports have also examined the impact of the sequences surrounding expansions on structure formation and stability.

### CUG Repeats

**Figure 1:**
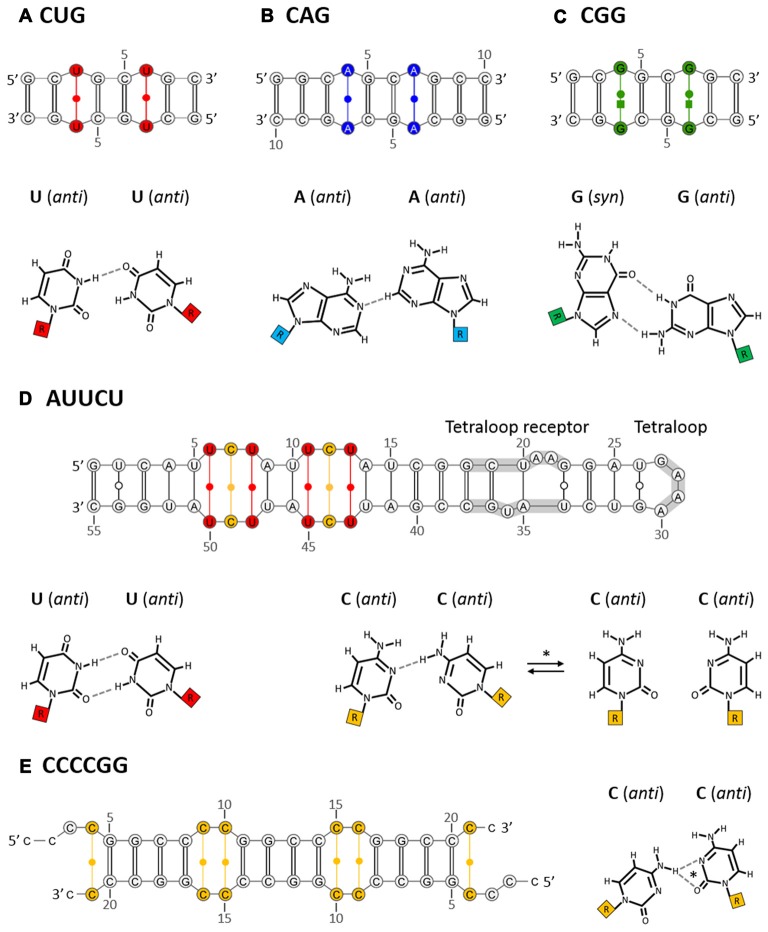
**Non-canonical base pairs in crystal structures of intermolecular duplexes formed by CUG (A)**, CAG **(B)**, CGG **(C)**, AUUCU** (D)** and CCCCGG** (E)** oligomers. The examples of duplexes that were analyzed by X-ray crystallography presumably representing the stem part of the corresponding hairpins (top panel) are shown. These duplexes contain standard Watson-Crick base pairs which are interrupted with non-canonical pairs specific for each repeated sequence (bottom panel, hydrogen bonds drawn with dashed lines). The secondary structures of crystalized RNAs are annotated according to the Leontis/Westhof nomenclature. Additionally, different colors represent different non-canonical base pairs: red, U-U; blue, A-A; green, G-G; orange, C-C. A crystallization-promoting tetraloop/tetraloop receptor motif that aided crystallization of AUUCU repeat RNA is indicated with gray underline. The secondary structures of duplexes and non-canonical base pairs specific for each repeated motif are described in the text in details. *In the case of AUUCU repeats the non-canonical C-C base pair can form either one- or non-hydrogen bond geometries. On the other hand, one of two possible one-hydrogen bond geometries characterize non-canonical C-C base pairs in CCCCGG repeat RNA.

To establish whether isolated CUG repeats and other trinucleotide repeats (TNRs) adopt higher-order RNA structures, two comparative studies were performed (Sobczak et al., 2003, 2010). First, using chemical (Pb^2+^ ions) and enzymatic (S1 nuclease, T1, T2 and V1 ribonucleases) cleavages, the structures of CCUG, AAG and all CNG repeat motifs (N = A, C, G or U) in solution were analyzed (Sobczak et al., 2003). In that study, a CUG motif repeated 17 times was shown to form hairpin structures composed of a stem with periodically occurring standard C-G and G-C base pairs and a single periodic U-U base pair whose nature was further examined by X-ray crystallography (Figure 1A; Mooers et al., 2005; Kiliszek et al., 2009). The terminal loop of this hairpin was composed of four nucleotides. Moreover, these CUG repeats formed several alternative, “in register” alignments, i.e., “slippery hairpins”. These hairpin variants differed in the lengths of their protruding 3′ ends. By using CUG repeat RNAs with end sequences that form stable GC-clamps, the “slippage effect” could be eliminated to produce a single CUG hairpin alignment. When an even number of the CUG repeats is clamped, a 4-nt terminal loop forms; however, 3-nt loops are present with an odd number of repeats, thus illustrating the influence of the sequences flanking the CUG repeats (and other TNRs) on the structural features and biological properties of these motifs.

More recently, a comprehensive structural study of a complete set of 20 TNRs that were repeated 17 times was carried out using a set of chemical (Pb^2+^ ions) and enzymatic (S1, Cl3, Mung bean nucleases; T1 and V1 ribonucleases) structure probing and biophysical methods (UV melting spectra, circular dichroism (CD) spectra and gel mobility analysis). As a result, TNRs have been grouped into four different structural classes: (1) unstructured RNAs; (2) semistable hairpins; (3) fairly stable hairpins; and (4) very stable G-quadruplexes. In agreement with previously described studies, CUG repeat motifs (together with CAA, CGU and other three CNG motifs) form fairly stable hairpins (Sobczak et al., 2010). In the same work, the thermodynamic stability of CNG repeats was further assessed by UV-monitored structure melting experiments (Sobczak et al., 2010). Among all TNRs which are implicated in Triplet Repeat Expansion Diseases (TREDs), the CUG motif has been shown to be the least thermodynamically stable, regardless of whether the measurements were performed in the presence of Na^+^ or K^+^ ions. The order of stability, starting from the most stable repeat, is as follows: CGG, CAG, CUG and CCG (in 100 mM NaCl) or CAG, CGG, CUG and CCG (in 100 mM KCl; Broda et al., 2005). Other calorimetric and structural (UV melting and/or CD spectroscopy) studies that also included CUG repeats have been performed (Pinheiro et al., 2002). Moreover, the hairpin structure formed by isolated, expanded CUG repeats (CUG_136_) has been visualized using electron microscopy (Yuan et al., 2007).

**Figure 2:**
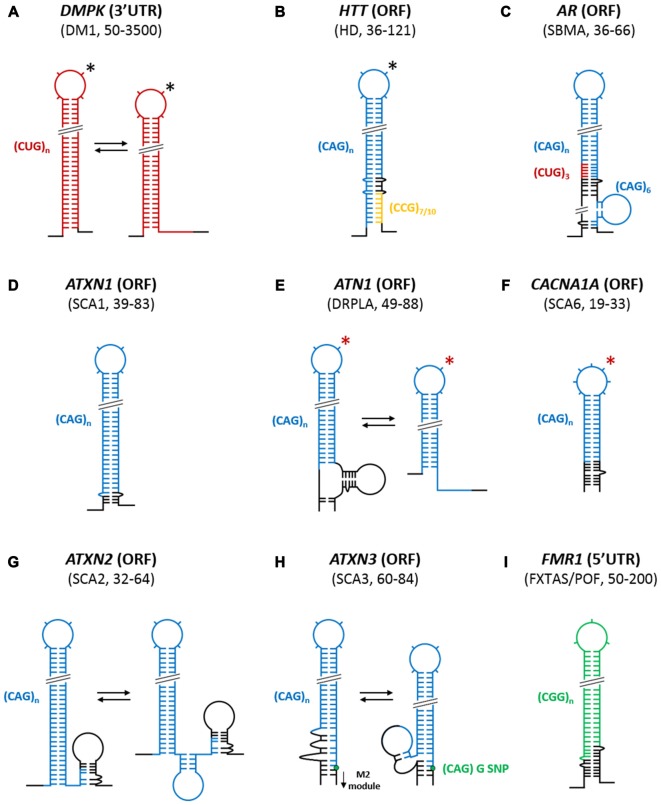
**Structural organization of trinucleotide repeats (TNRs) in the context of the flanking sequences of different triplet repeat expansion diseases (TREDs) transcripts.** Based on chemical and enzymatic RNA structure probing/(experimental RNA structure probing) simplified models of uninterrupted, mutant (transcripts) dystrophia myotonica protein kinase *(DMPK)* **(A)**, huntingtin (*HTT*) **(B)**, androgen receptor *(AR)* **(C)**, *ATXN1* **(D)**, *ATN1* **(E)**, *CACNA1A* **(F)**, *ATXN2* **(G)**, *ATXN3* **(H)** and fragile X mental retardation 1 *(FMR1)* **(I)** transcripts fragments implicated in a range of different TREDs are depicted. In some TREDs-related transcripts, the repeats themselves form autonomous hairpin structures that show slippage tendencies (*DMPK, ATN1, ATXN2 and ATXN3*). In several other transcripts, sequences flanking the repeat (*ATXN1*, *CACNA1A* and *FMR1*) influence the stability of the structure leading to the fixation of a single conformation. In *HTT* and *AR* transcripts, two different repeat sequences CCG and CUG respectively together with flanking sequences form a single composite hairpin structure. The localization of mutated repeat tracts within their corresponding transcripts (5′UTR, ORF or 3′UTR), characteristic pathogenic number of repeats (range) and implicated neurodegenerative diseases are also indicated. Different colors represent different repeated sequences: red, CUG; blue, CAG; green, CGG; orange, CCG; and black, specific flanking sequences. *The predicted secondary structures of the mutant transcripts that were not experimentally confirmed, but their normal transcript structure was established, are indicated by an red asterisk. The number of protrusions in the TNR hairpin terminal loops correspond to the number of nucleotides in these loops when odd number of repeats is studied. *In the case of *DMPK* and *HTT* transcripts where enlargement of 4-nt terminal loop into an alternative 7-nt terminal loop variant as the result of slippage tendencies (effects) of these TNRs, is also observed (black asterisks). Green node represent the guanosine in the (CAG)G/C polymorphism in *ATXN3* transcript.

Thus far, only one study has provided structural insights into the CUG repeat region from the 3′UTR of the *DMPK* transcript, which is involved in DM1 pathogenesis (Figure 2A; Napierala and Krzyzosiak, 1997). This study was the first of a series of experiments that began to probe CNG repeat structures in a wider transcript context. The analysis was performed using chemical (Pb^2+^ ions) and enzymatic (S1 and T1 nucleases) structure probing of *in vitro*-transcribed RNAs containing increasing lengths of CUG repeats (5, 11, 21 and 49) together with flanking sequences (30 and 35 nucleotides at the 5′ side and 3′ side of the CUG repeat, respectively). The analysis demonstrated that five repeats, which is the most common, non-pathogenic number of repeats in the population, do not form any secondary structures. Upon increasing the length of the CUG repeats, the stability of the formed structures increased: 11 repeats formed unstable hairpins, 21 repeats formed semi-stable hairpins, and the expanded 49 CUG repeats formed fairly stable hairpins. Moreover, as the sequences flanking the repeats did not “freeze” the repeat alignment, thus resulting in alternative structures, the CUG repeat hairpins are referred to as “slippery” (Figure 2A). Furthermore, increasing the CUG repeat length from, e.g., 21–49 repeats caused the increases in the length and stiffness of the repeat hairpin stem and appeared to enlarge the hairpin terminal loop (Napierala and Krzyzosiak, 1997).

By using X-ray crystallography, a number of studies have revealed the detailed structures of short CUG-containing oligoribonucleotides (Mooers et al., 2005; Kiliszek et al., 2009; Kumar et al., 2011; Coonrod et al., 2012; Tamjar et al., 2012). When crystalized, these oligomers (up to six CUG repeats) pile onto each other to form an intermolecular duplex that presumably represents the stem part of the hairpin, which is formed by long CUG repeats (Figure 1A). Furthermore, these studies showed that RNA is double helical and has general features of the A form, in which two standard C-G and G-C base pairs are interrupted by a non-canonical U-U pair. While standard Watson-Crick pairing between C-G and G-C stabilizes duplex formation, the occurrence of the periodic U-U pair contributes to the unique geometry of the helix. Unlike in most of the previously observed U-U pairs that had two hydrogen bonds, the U-U pairs in the CUG repeat context appear to be “stretched” (“stretched U-U wobble”) and to form only a single direct hydrogen bond between the N3 imino group of one uracil residue and the carbonyl O4 atom of the other (Figure 1A). In this CUG repeat duplex, the two opposing uracil residues do not create any accommodation problem because they remain well separated from each other; thus, the disruption of the overall helix structure is low (Kiliszek et al., 2009).

### CAG Repeats

By using both enzymatic and chemical RNA structure probing experiments, it was initially shown that isolated CAG repeats (CAG_17_), similar to the previously described CUG repeats, form hairpin structures in solution, with a stem composed of periodically occurring standard C-G and G-C base pairs that are divided by a single, periodic non-canonical A-A pair (Sobczak et al., 2003). These CAG repeat hairpins were shown to form several alternative alignments (“slipped hairpins”) that differ in the lengths of their protruding 3′ ends. When GC-clamps were added at both ends of the CAG repeat sequence, the slippage effect was abolished. Moreover, in this clamped configuration, a CAG repeat hairpin composed of an even number of repeats (CAG_16_) forms a 4-nt apical loop. However, when an odd-numbered hairpin was studied (CAG_17_), a larger 7-nt loop appeared (Sobczak et al., 2003). In another more robust study (a complete set of TNRs was assayed), it was shown that the CAG repeats (CAG_17 or 20_) form fairly stable hairpins (3rd structural class, similar to CNG, CAA and CGU motifs; Sobczak et al., 2010). Moreover, UV-monitored structure melting experiments have shown that among all TREDs-related repeats, the CAG repeat motif is the most thermodynamically stable under K^+^ ion conditions (second to the CGG motif under Na^+^ ion conditions; Broda et al., 2005; Sobczak et al., 2010).

In recent years, using X-ray crystallographic methods, the structures of several oligoribonucleotides composed of CAG repeats have been reported (Kiliszek et al., 2010; Yildirim et al., 2013). These short RNA fragments stack onto each other, forming intermolecular duplexes that represent the stem portions of CAG repeat hairpins (Figure 1B). Furthermore, these duplexes acquire the general characteristics of RNA-A helices, where the non-canonical, periodic A-A base pairs are settled between the canonical C-G and G-C pairs that play a stabilizing role. As the positioning of two bulky adenine rings opposite each other within the helical structure seems to be “sterically challenging”, local, moderate disruption of the helix geometry, resulting in local unwinding of the helical structure and subsequent widening of the major groove, was observed (Kiliszek et al., 2010). In this CAG repeat duplex, both of the opposite adenine residues are in the *anti* conformation and are shifted out of the helical axis towards the major grove to avoid collision. The adenine residue, which plays a role as a H-bond donor, is shifted more, thus resulting in a “thumbs up” conformation. Furthermore, the A-A pairs in the CAG repeat context form only a single, unusual, weak hydrogen bond between the carbon atom C2-H2 of one adenine residue and the nitrogen N1 atom of the other (C2-H2⋅⋅⋅N1 hydrogen bond) (Figure 1B; Kiliszek et al., 2010). This type of A-A wobble pairing has not been previously reported. In a more recent study, by using X-ray crystallography, nuclear magnetic resonance (NMR) spectroscopy and molecular dynamics (MD) simulation analysis, different nature of the non-canonical A-A base pairs has been shown (Tawani and Kumar, 2015). In the analyzed model RNA duplex containing three CAG repeats with additional flanking sequences, conformational dynamics were suggested due to the specific hydrogen bonding pattern and stacking interactions of the non-canonical A-A base pairs. Unlike the previous reports, one of the closing A-A base pairs showed *syn*-*anti* conformation with one hydrogen bond between the *exo*-amino group of A(*syn*) and N1 atom of the A(*anti*) (N6H⋅⋅⋅N1 hydrogen bond). Moreover, the second closing A-A base pair, as well as, the A-A base pair located in the center of the duplex all had *anti*-*anti* conformations with no hydrogen bonds (Tawani and Kumar, 2015). As poor and ambiguous electron density maps were analyzed in this study it is still a matter of a debate whether different, dynamic A-A base pair conformations truly exist within CAG repeats.

Until now, the structures formed by CAG repeats in their native, transcript context are the most extensively studied TNRs. These studies focused on assessing whether specific sequences flanking the CAG repeats contribute to the overall characteristics of the CAG hairpin structures, i.e., the formation of the multiple alignment hairpins (“slipped” hairpin) or a single alignment hairpin (“frozen” hairpin; Michlewski and Krzyzosiak, 2004; Sobczak and Krzyzosiak, 2004a, 2005; de Mezer et al., 2011). Moreover, the influence of naturally occurring repeat interruptions on the structures formed by CAG repeats was also investigated. These structural studies were mainly performed using both chemical (Pb^2+^) and enzymatic RNA structure probing experiments with the use of a battery of probing reagents, e.g., S1, Mung Bean nuclease and RNases T1, T2, V1 and H.

In another study, the unique, complex architectures of the hairpins formed by CAG repeats present in *HTT* and androgen receptor (*AR*) transcripts, which are affected by both the specific flanking sequences and another type of neighboring, triplet repeats, have been established (de Mezer et al., 2011). As shown in Figure 2B, in the *HTT* transcripts expansion-prone CAG repeats [(CAG)_n_] have polymorphic CCG repeat tract [(CCG)_7 or 10 nt_] at their 3′ side that is separated by a 12-nucleotide specific sequence. This specific neighborhood causes the HTT CAG repeat variants to have a tripartite modular structure composed of the base, which is the most stable part of the stem and where the 5′ part of the CAG repeats are engaged in base pairing with CCGs; the central module, which is formed by the partially base pairing CAG repeats with 12-nucleotide specific sequence; and the terminal section, which is part of the hairpin structure and which is composed exclusively of CAG repeats. The difference between normal and mutant *HTT* transcripts is only restricted to the terminal section, which in the mutant transcript gets elongated, while the other structural modules remain the same. As the stability of the base module consisting of base-paired CAG and CCG repeats is much higher than other hairpin modules, the latter may form alternative structural variants resulting from the slippage effect of the CAG repeats (de Mezer et al., 2011). More recently, the structures of CAG repeats of normal and pathogenic length (*HTT* transcript), in the context of the entire first exon (harboring the repeats) and the neighboring 5′UTR sequence, have been investigated by *in vitro* selective 2′-hydroxyl acetylation analyzed by primer extension (SHAPE) analysis (Busan and Weeks, 2013). In addition to the observation made by de Mezer et al. (2011), additional base pairing between a few CAG repeats from the 5′ side with nucleotides in the 5′UTR sequence was observed. Moreover, it was shown that when a normal number of CAG repeats was studied, the CAG hairpin was either absent (CAG_17_) or short (CAG_23_; Busan and Weeks, 2013).

In the case of *AR* transcript, which is involved in spinal and bulbar muscular atrophy (SBMA), not one but two different types of neighboring, triplet repeats, together with specific flanking sequences, contribute to the formation of a composite CAG repeat hairpin structure (de Mezer et al., 2011). As shown in Figure 2C, the *AR* transcript contains three monomorphic CUG repeats [(CUG)_3_] directly upstream of a polymorphic, expansion-prone CAG repeat tract [(CAG)_n_]. Moreover, six monomorphic CAG repeats [(CAG)_6_] are located downstream of the CAG repeat tract and are separated by an 18-nucleotide specific sequence. As presented in Figure 2C, the monomorphic (CUG)_3_ repeats fully base pair with the last three CAG repeats of the (CAG)_n_ tract to form a strong 12-bp stabilizing clamp, which is responsible for the presence of both normal and mutant *AR* transcripts in only the “frozen” type of hairpin form with a single RNA alignment. In addition, further stabilization of the repeat hairpin is conferred by the base-pairing system of the nearest specific CAG flanking sequences. Moreover, the monomorphic (CAG)_6_ repeat tract, which is located 18 nt downstream from the polymorphic, expansion-prone CAG repeat tract [(CAG)_n_], is not involved in the formation of the long repeat hairpin structure, but rather, forms an autonomous short hairpin. The only difference between the normal and mutant *AR* transcript is the length of the CAG repeat hairpin, which is formed solely by polymorphic expansion-prone CAG repeats and which contains either a 4- or 7-nt terminal loop, depending on the CAG repeat number according to the rule observed for isolated, clamped, CAG repeat structures (Sobczak et al., 2003).

Structural studies of the CAG repeat regions from the *ATXN1* (Sobczak and Krzyzosiak, 2004a) and the calcium voltage-gated channel subunit alpha1 A (*CACNA1A*) transcripts (Michlewski and Krzyzosiak, 2004), which are implicated in SCA1 and SCA6, respectively, have also revealed the importance of the specific flanking sequences in stabilizing CAG repeat hairpin structures (Figures 2D,F). In both transcripts, there is a strong, naturally occurring clamp that is formed by the specific flanking sequences at the base of the repeat hairpin. For *ATXN1*, the clamp consists of a perfectly matching 6-bp long fragment. These clamps cause the CAG repeats to “freeze” in a single alignment and to form stable hairpins with terminal loops of different sizes depending on the repeat number (4-nt or 7-nt loops). The stem structure and loop size follow the pattern observed for model of isolated CAG repeats containing a GC clamp (Sobczak et al., 2003).

Using similar structure probing approaches, several “slipped” hairpin variants have been observed for CAG repeats of the atrophin (*ATN1*) (Michlewski and Krzyzosiak, 2004), *ATXN2* (Sobczak and Krzyzosiak, 2005), and *ATXN3* (Michlewski and Krzyzosiak, 2004) transcripts, which are implicated in dentatorubral-pallidoluysian atrophy (DRPLA), SCA2 and SCA3, respectively. In *ATN1* and *ATXN2* transcripts (Figures 2E,G), their specific repeat flanking sequences do not stabilize the CAG repeat structure, as no clamping of the hairpin by flanking sequences is observed. Furthermore, the “slipped” *ATN1* hairpins have the repeats moved towards their 3′ end and contain a 4-nt terminal loop, as observed for unclamped CAG model transcripts (Figure 2E; Sobczak et al., 2003). In contrast, in the *ATXN2* transcript, the specific flanking sequences located at the 3′ side of the CAG repeat tract interact with 3′ terminal CAG repeats, which results in a reduction of the CAG repeat stem length. This interaction, however, does not force single alignment of the uninterrupted CAG repeats in the *ATXN2* mutant transcript because several “slippery” hairpin variants are observed (Figure 2G). For the *ATXN3* transcript, the CAG repeat region has a particular architecture that is greatly influenced by both 3′- and 5′-specific flanking sequences (Figure 2H). First, several 3′ terminal CAG repeats are involved in a quasi-stable interaction with the 18-nt pseudo-repeat sequence that flanks the CAG repeats on its 5′ side. Moreover, the (CAG)C/G single-nucleotide polymorphism (SNP) that is located between CAG repeat tract and its specific 3′ flanking sequence does influence the structure of both of these sequences. Depending on which SNP variant is present, the size of the CAG hairpin terminal loop and, to a minor extent, the structure formed by the 3′ flanking sequence (M2 module) change. Furthermore, the CAG repeat hairpin forms several alternatively aligned variants that are “slipped” towards the 5′ end, in contrast to the unclamped CAG repeats in model transcripts due to the presence of the 18-nt pseudo-repeat sequence.

**Figure 3:**
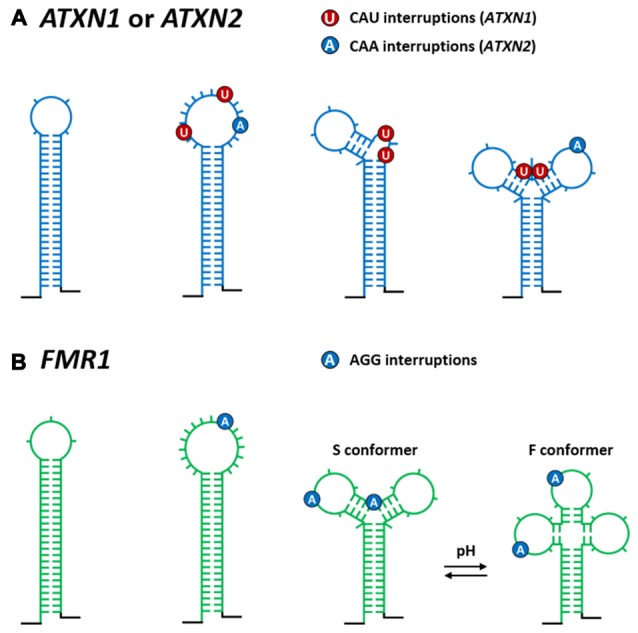
**Influence of repeat interruptions on structures formed by normal repeat length TNRs in different TREDs transcripts. (A)** The effect of CAU and CAA interruptions on the CAG hairpin structures in *ATXN1* and *ATXN2* transcripts respectively, and **(B)** the effect of AGG interruptions on the CGG hairpin in *FMR1* transcript. Both the number and the location of the interruptions determine the structure of the repeat tract region. Depending on how the interruptions are organized in transcripts they can widen the existing terminal loop, nucleate out additional loops, split the sequence into two separate hairpins or form specific branched structures in which the interruptions are predominantly present in terminal and internal hairpin loops. In case of interrupted CGG repeats, a pH-dependent mechanism that causes the Y-shape structure to adopt different alternative conformers (S and F conformer) is shown.

By using comprehensive population genotyping surveys, specific interruptions CAT and CAA were found within CAG repeat tracts of *ATXN1* and *ATXN2* alleles, respectively (Sobczak and Krzyzosiak, 2004b; Rozanska et al., 2007). Interestingly, these types of repeat interruptions (usually 1–3) were observed in normal, but not in the expanded, mutant CAG repeat tracts and therefore are believed to function on the genomic level as protective elements, preventing further repeat expansion during maternal transmission and development (Pearson et al., 1998). Additionally, the effects of CAU and CAA interruptions on the CAG hairpin structures in *ATXN1* (Sobczak and Krzyzosiak, 2004a) and *ATXN2* (Sobczak and Krzyzosiak, 2005) transcripts have been shown. As presented in Figure 3A, both the number and localization of the interruptions determine the structure of this region in both the transcripts. Most commonly, in the *ATXN1* and *ATXN2* transcripts, one or two CAU triplets (approximately 90% in populations) and two CAA triplets (78%–95% in populations), respectively, were shown to destabilize the stem of the single, long CAG repeat hairpin. If two interruptions break the regularity of the CAG repeat tracts, they are always separated by one CAG repeat in the *ATXN1* transcripts and by four CAG repeats in the *ATXN2* transcripts. Depending on how the CAU and CAA interruptions are organized in transcripts, they can widen the existing terminal loop, nucleate out additional loops, split the sequence into two separate hairpins (mainly in *ATXN1* transcripts), or form specific branched structures with the interruptions localized in terminal loop/s (mainly in *ATXN1* transcripts; Figure 3A). As these effects ultimately lead to the shortening of the single long CAG hairpin structure, it is hypothesized (Sobczak and Krzyzosiak, 2004a, 2005) that both CAU and CAA triplets can delay disease onset or severity (Matsuyama et al., 1999; Tian et al., 2000; Peel et al., 2001; Hussey et al., 2002). These observations might be the consequence of the reduced, diseased-causing sequestration of RNA-binding proteins by interrupted CAG repeat tracts.

### CGG Repeats

Similar to the other CNG repeats, the higher-order RNA structures that were formed in solution by isolated CGG repeat motifs (CGG_17_) were found to form hairpins with the stem composed of periodically occurring C-G and G-C base pairs that are separated by a single non-canonical G-G pair (Sobczak et al., 2003). In contrast to the other CNG repeats, the structure of the CGG hairpin is more rigid and does not form “in register” conformations, i.e., “slippery hairpins” (Sobczak et al., 2003). When the CGG repeat hairpin was GC-clamped and had an even number of repeats (CGG_16_), a 4-nt apical loop was formed. In the case of odd-numbered CGG repeat hairpin (CGG_17_) a tighter 3-nt terminal loop was observed (Sobczak et al., 2003). In a more recent, comprehensive, RNA structure probing study, it was shown that isolated CGG motifs (CGG_17 or 20_), together with other three CNG, CAA and CGU motifs, form fairly stable hairpins (3rd structural class; Sobczak et al., 2010). Moreover, calorimetric studies revealed that among all CNG repeats, CGG repeat motif is the most thermodynamically stable in Na^+^ ions conditions (second to CAG motif in K^+^ ions conditions; Broda et al., 2005; Sobczak et al., 2010). Other biophysical (NMR and UV spectroscopy—Zumwalt et al., 2007) and biochemical (gel mobility analysis—Khateb et al., 2004; Ofer et al., 2009) studies on CGG repeat motifs have also been performed.

In recent years, the X-ray crystal structures of CGG-containing oligoribonucleotides have been published (Kiliszek et al., 2011; Kumar et al., 2011). The analyzed CGG oligomers [G(CGG)_2_C] formed intermolecular duplexes representing the stem portions of the CGG repeat hairpin (Figure 1C). Furthermore, the identified helices retained an A form, where the non-canonical G-G pairs were flanked by canonical stabilizing C-G and G-C pairs. The steric hindrance caused by two bulky guanine residues opposite each other within the helical structure is resolved by having one guanosine in the *syn* conformation and the other in the *anti* conformation (Figure 1C). This *syn*-*anti* arrangement causes the local, moderate, unwinding of the helix structure and widening of the major groove. In all G-G pairs, two direct hydrogen bonds are formed between the O6, N1 atoms from the G(*syn*) and N7, N2H atoms from the G(*anti*), respectively (O6⋅⋅⋅N1H and N7⋅⋅⋅N2H) (Kiliszek et al., 2011). This kind of interaction between a wobble G-G pair has been commonly observed in other NMR and crystallographic RNA structures. The observed strong hydrogen bonding system could explain why the model CGG repeat transcripts do not show the slippage effect, as opposed to the less stable CAG and CUG repeats (Sobczak et al., 2003).

**Figure 4:**
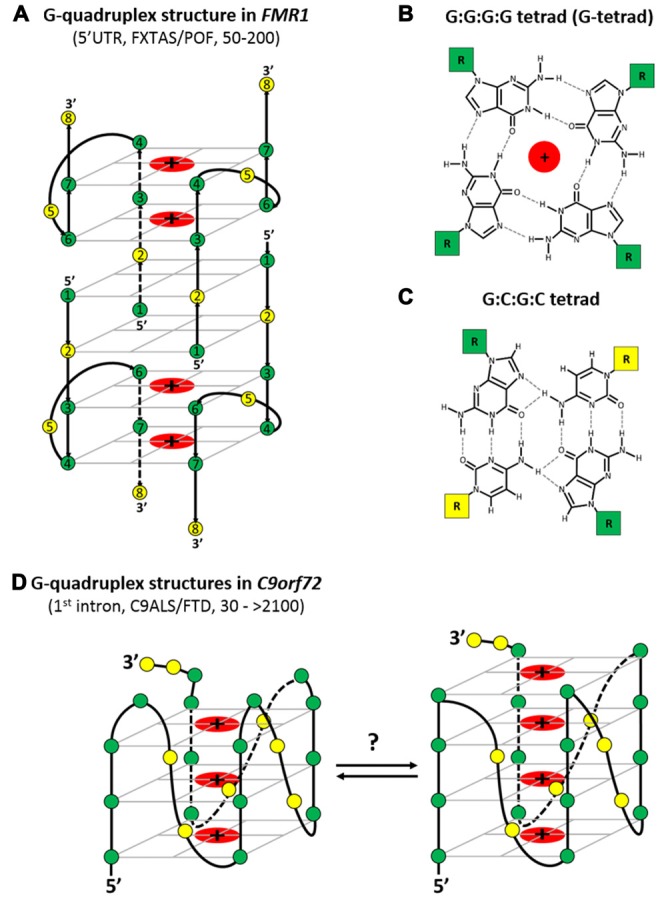
**The G-quadruplex structures formed by CGG and GGGGCC repeats found in *FMR1* and *C9orf72* transcripts, respectively (implicated in fragile X-associated tremor ataxia syndrome (FXTAS)/premature ovarian failure (POF) and *C9orf72* amyotrophic lateral sclerosis and frontotemporal dementia (C9ALS/FTD) pathogenesis respectively). (A)** Schematic representation of the symmetrical, tetramolecular G-quadruplex structure formed *in vitro* by short CGG-containing oligoribonucleotides [G(CGG)_2_C]. This G-quadruplex structure is composed of two mixed, antiparallel G:C:G:C tetrads devided by parallel G:G:G:G tetrad planes.** (B)** Schematic representation of the proposed symmetrical G-quadruplex structures formed by GGGGCC repeats, which adopt a parallel topology and consist of three or/and four stacks of parallel G:G:G:G tetrad planes. **(C,D)** Schematic outline of the hydrogen bonding status (*drawn with dashed lines*) between four guanines in G-tetrad **(C)** and between two guanines and two cytosines in a mixed G:C:G:C tetrad **(D)**. The potassium or sodium ions that were shown to stabilize the G-quadruplex structures formed by CGG and GGGGCC repeats are shown as (+) symbol. The localization of both CGG and GGGGCC repeat tract within corresponding transcripts, pathogenic number of repeat and implicated neurodegenerative diseases are also indicated. Green nodes—guanine residues; yellow nodes—cytosine residues.

The exact structural properties of transcripts containing CGG repeats have been a matter of debate. While strong biophysical and biochemical evidence for CGG duplex and hairpin structures has been presented (Sobczak et al., 2003, 2010; Napierala et al., 2005; Zumwalt et al., 2007; Kiliszek et al., 2011; Kumar et al., 2011), other higher-order structures, i.e., quadruplexes, have also been observed (Handa et al., 2003; Khateb et al., 2004, 2007; Ofer et al., 2009; Malgowska et al., 2014; Gudanis et al., 2016). Recently, by using a battery of biophysical methods, such as UV-visible, CD and NMR spectroscopies, electrospray ionization mass spectrometry (ESI-MS), small-angle X-ray scattering (SAXS) and native gel electrophoresis analysis, two different quadruplex structures that short CGG-containing oligoribonucleotides formed in solution were identified (Malgowska et al., 2014; Gudanis et al., 2016). Figure 4A shows the specific G-quadruplex structure that is formed by short CGG-containing oligoribonucleotides [G(CGG)_2_C] in the presence of K^+^ ions (Malgowska et al., 2014). As demonstrated, this symmetrical, tetramolecular G-quadruplex structure is composed of two mixed, antiparallel G:C:G:C tetrads divided by parallel G:G:G:G tetrad (G-tetrad) planes (Figures 4B,C). However, the structures formed by both G(CGG)_2_C and G(CGG)_4_C oligomers in solution are highly polymorphic, and other type of structures can be adopted depending on the presence of different cations (K^+^, Na^+^ or NH_4_^+^), the length of the repeats and the concentration of RNA used. For example, when the G(CGG)_2_C oligomer is studied in Na^+^ solutions, it maintains a balance between G-quadruplex/duplex structures, but when it is studied in HeLa cellular extracts where K^+^ ions are prevalent, the G(CGG)_2_C oligomer almost exclusively forms duplexes. Recently, a novel quadruplex architecture formed by the 8-bromoguanosine-modified molecule GC^Br^GGCGGC was characterized (Gudanis et al., 2016). This unnatural modification locks the ^Br^G:G pairs exclusively in favorable ^Br^G(*syn*)-G(*anti*) conformations, thus increasing the thermodynamic stability and the homogeneity of the RNA structures that formed. As the folding rules of both the quadruplexes formed by CGG repeats are yet unclear and may depend on other factors, it is of great interest to study these structures further.

Thus far, two studies have been carried out to provide structural insights into the CGG repeat region from the 5′UTR of the *FMR1* transcript, which is associated with a range of different clinical phenotypes (e.g., FXTAS and premature ovarian failure (POF)) of increased severity depending on the extent of CGG tract expansion (Handa et al., 2003; Napierala et al., 2005). As seen in Figure 2I, uninterrupted CGG repeats form a hairpin that is composed of non-canonical G-G pairs flanked by stabilizing C-G and G-C pairs (with a 3- or 6-nt terminal loop) and that is further stabilized by base-paired 3′- and 5′-specific flanking sequences. Moreover, the 3′ end of the CGG repeats was shown to be engaged in base pairing with a section of the 3′ flanking sequence (Napierala et al., 2005) The majority of normal and premutation size *FMR1* alleles contain specific AGG interruptions (1–4, usually 2) in the CGG repeat tract. These AGG triplets were shown to destabilize the single long CGG hairpin structure in different ways (Figure 3B). Depending on their localization within the repeats, they can enlarge the existing terminal loop, nucleate out additional autonomous hairpin loops or form a Y-shaped structure. Regarding the latter, the polymorphic A lies in one of the double stranded arm and base pairs with the U residue located in the 3′ flanking sequence. Moreover, a pH-dependent mechanism that causes the Y-shaped structure to adopt different alternative conformers (S and F conformers) was identified (Figure 3B; Napierala et al., 2005). These AGG interruptions have been shown to function as protective elements that prevent CGG repeat expansion at the DNA level during maternal transmission (Eichler et al., 1994; Pearson et al., 1998; Dombrowski et al., 2002). Additionally, depending on the number and localization of the interruptions, it is hypothesized that they can protect some premutation carriers from FXTAS and POF by shortening the pathogenic length of hairpins composed of pure CGG repeats.

### AUUCU Repeats

To date, higher-order RNA structures formed by pentanucleotide AUUCU repeats, which are present in intron 9 of the *ATXN10* transcript implicated in SCA10, have been a subject of two structural studies (Handa et al., 2005; Park et al., 2015). By using a combination of enzymatic (S1 nuclease, RNase V1) RNA structure probing and biophysical (CD and NMR) approaches, it was demonstrated that as few as nine AUUCU repeats [(AUUCU)_9/11/14 or 17_] form an unusual RNA hairpin structure (Handa et al., 2005). The stem of this hairpin has characteristics of A-form geometry and, as revealed by NMR analysis, contains a mixture of A-U and U-U base pairing in a 1:1 ratio. This ratio suggests that the stem of the AUUCU repeat hairpin harbors ^5^′UCU^3^′/^3^’UCU^5^′ internal loops with two noncanonical U-U pairs and one noncanonical C-C pair, which are closed by two canonical A-U pairs (^5^′AU^3^′/^3^′UA^5^′ loop closing pairs) (Figure 1D; Handa et al., 2005). Recently, the structural characteristics of AUUCU repeat-containing RNAs have been confirmed, and further insights were gained using X-ray crystallography followed by a robust computational analysis of the structure via MD simulations (Park et al., 2015). In that study, a crystallization-promoting tetraloop/tetraloop receptor motif was utilized to aid crystallization of a model RNA containing two copies of ^5^′AUUCU^3^′/^3^′UCUUA^5^′ motifs (refined to 2.8 Å; Figure 1D). This analysis showed standard Watson-Crick base pairing in the ^5^′AU^3^′/^3^′UA^5^′ loop closing pairs, thereby stabilizing the AUUCU repeat duplex/hairpin structure. For the hydrogen bonding status of the internal ^5^′UCU^3^′/^3^’UCU^5^′ loops, a more complex and dynamic transitions were suggested. According to the X-ray data, the opposite uracil residues constituting both noncanonical U-U pairs are in the *anti* conformation and possess two hydrogen bond geometries, where the carbonyl O4 atom and the N3 amino group of the first U form two hydrogen bonds with the N3 amino group and carbonyl O2 atom of the second U, respectively (O4⋅⋅⋅N3H; N3H⋅⋅⋅O2) (Figure 1D). In the case of the central noncanonical C-C base pair, two different conformations were suggested: (1) a frequent, one hydrogen bond geometry, where both cytosine residues are in the *anti* conformation and form one very weak hydrogen bond between the exo-amino group of one C residue and the N3 atom of the other C residue (N4H⋅⋅⋅N3); and (2) a stable zero hydrogen bond conformation, which is stabilized by a specific hydration pattern, as suggested by *in silico* computational analysis. Moreover, it is hypothesized that these extremely weak and dynamic C-C non-canonical pairing interactions as well as the disrupted base stacking of the C-C pair with the neighboring U-U pairs cause the internal ^5^′UCU^3^′/^3^’UCU^5^′ loops to be the least thermodynamically stable elements of the AUUCU repeat hairpin structure. Furthermore, as proposed by the MD simulation analysis, this dynamic feature of the internal loops affects the overall AUUCU A-form helix stability, causing its rearrangement into single-stranded conformations (quasistable hairpin structures). This ^5^′UCU^3^′/^3^’UCU^5^′ loop is thought to be a site where RNA unwinding starts.

### GGGGCC/CCCCGG Repeats

In recent years, numerous structural and functional studies have been carried out to understand RNA toxicity in newly described hexanucleotide repeat expansion which is implicated in C9ALS/FTD. In these diseases, the expanded repeats are transcribed bidirectionally, generating noncoding sense (GGGGCC)_n_ and antisense (CCCCGG)_n_ transcripts that both form different higher-order RNA structures.

Initially, Fratta et al. (2012) used biophysical approaches (NMR and CD spectroscopies) to confirm the results of an *in silico* computational analysis performed with a G-quadruplex prediction tool (QGRS mapper) and showed that an isolated (GGGGCC)_3_GGGGC RNA oligomer (C9Gru) is minimally required for the formation of a specific G-quadruplex structure (Fratta et al., 2012). As shown in Figure 4D, this highly stable G-quadruplex structure adopts a parallel topology (typical for most other RNA G-quadruplexes) and consists of four stacks of parallel G:G:G:G tetrad (G-tetrad) planes. In each G-tetrad plane, guanine bases are arranged in a square cyclic pattern connected by eight Hoogsteen hydrogen bonds and are arranged in a planar configuration around a central monovalent metal cation (interacting with guanine O6 atoms) that significantly affects G-quadruplex stability and topology (Figure 4B). The four stacked G-tetrads, which are bound with metal ions positioned centrally and phosphate backbones positioned laterally, are connected through a propeller loop arrangement composed of two cytosines, which ensures parallel topology.

Some deviations from the above G-quadruplex structure have also been observed. As suggested by Reddy et al. (2013) and Haeusler et al. (2014), the G-quadruplex structure consists of three, not four, G-tetrad planes (Reddy et al., 2013; Haeusler et al., 2014). By using an RNase protection assay (RNase T1, which cleaves ssRNA at the 3′ end of guanine residues), it was shown that a model (GGGGCC)_4_ RNA in 100 mM KCl formed a symmetrical, parallel G-quadruplex structure almost exclusively, and the digestion pattern revealed a three-stacked G-tetrad plane topology, with guanine and two cytosines in the single-stranded loop region (not involved in the formation of the G-quadruplex structure) that connects laterally G-quadruplex phosphate backbones (Figure 4D).

Whether the formation of G-quadruplexes results from the association of GGGGCC repeats of the same *C9orf72* transcript (intramolecular G-quadruplexes) or from the interaction of GGGGCC repeats from different *C9orf72* molecules (intermolecular G-quadruplexes) is still a matter of a debate (Fratta et al., 2012; Reddy et al., 2013). It was suggested by CD spectroscopy that an isolated, (very short) model (GGGGCC)_3_GGGGC transcript (minimal C9Gru) can form stable G-quadruplex structures only through an intramolecular association (Fratta et al., 2012). It was suggested by CD spectroscopy and native PAGE analysis that an isolated, model (GGGGCC)_4_ transcript can form an extremely stable intramolecular G-quadruplex structure (Reddy et al., 2013) Additionally, by native PAGE, it was shown that the heterogeneity of the formed G-quadruplexes increases as the repeat length or RNA concentration increases. In contrast to (GGGGCC)_4_ RNA, additional slower migrating species were observed for (GGGGCC)_6 or 8_, which is consistent with the formation of additional intermolecular G-quadruplexes (multimeric). Moreover, such an increase in heterogeneity was observed, as both additional intramolecular and intermolecular G-quadruplexes were formed when a native 5′ flanking sequence (15-nt long) from *C9orf72* RNA was present upstream of the GGGGCC repeat tract. Hairpin formation may compete with or contribute to the formation of G-quadruplexes or other structures (Reddy et al., 2013). By using a battery of biochemical approaches (enzymatic structure probing (RNase A, T1, A/T1), UV crosslinking, and native and denaturing PAGE), it was demonstrated that an isolated, model (GGGGCC)_10_ RNA (oligomer) forms stable multimeric G-quadruplex structures in U87 nuclear extracts (*in vitro*, Conlon et al., 2016). These analyses showed two major, distinct G-quadruplex states resulting from 10 GGGGCC repeat transcripts that were differentially folded: one in which four GGGGCC repeats form one G-quadruplex and the other major conformation in which eight consecutive GGGGCC repeats form two G-quadruplex structures.

By using CD spectroscopy (Fratta et al., 2012; Reddy et al., 2013; Haeusler et al., 2014) and native PAGE (Reddy et al., 2013; Haeusler et al., 2014) analysis, it was shown that similar to other RNA G-quadruplexes, the folding of isolated model (GGGGCC)_n_ repeat RNAs (*n* = ~4) into a stable G-quadruplex structure is strongly influenced by the presence of monovalent cations (K^+^ promotes stable folding over Na^+^ and Li^+^ ions). Furthermore, at a physiological K^+^ ion concentration (pH), these structures were shown to be extremely thermodynamically stable (up to 95°C; Fratta et al., 2012; Reddy et al., 2013). Additionally, by using CD spectroscopy and RNase protection assay (RNase T1) analyses, it was demonstrated that an isolated model (GGGGCC)_4_ RNA oligomer can adopt either a G-quadruplex structure (almost exclusively) or a structure consistent with single-stranded bulges and hairpin conformations, depending on the presence or absence of K^+^ ions, respectively (Haeusler et al., 2014).

The G-quadruplex structures formed by GGGGCC repeats have only recently been observed in cells (Conlon et al., 2016). By using immunofluorescence (IF) microscopy and a G-quadruplex recognizing antibody (BG-4), it was shown that G-quadruplexes formed by the expanded GGGGCC repeats in mutant *C9orf72* RNAs exist and are components of pathogenic RNA foci observed in fibroblasts and astrocytes derived from C9ALS/FTD patients; they are also major components of insoluble protein/RNA aggregates that have been isolated from disease-relevant regions of post-mortem c9ALS brains. However, the nature of these G-quadruplexes could not be established, as BG4 does not differentiate between any particular G-quadruplex conformation (parallel, anti-parallel, mixed parallel/anti-parallel, intermolecular or intramolecular; Conlon et al., 2016). Recently, the formation of G-quadruplex structures in mammalian transcriptome was analyzed with a modified, high-throughput RNA chemical probing techniques—DMS-seq and SHAPE-seq (Guo and Bartel, 2016). By applying these methods to human transcriptome the authors were able to identify thousands (>10,000) of novel G-quadruplex structures *in vitro*. However, when studied in cells (*in vivo*), these G-quadruplex forming regions were shown to be globally unfolded, presumably by a robust and effective machinery consisting of unknown RNA helicases and other ssRNA-binding proteins, that await to be fully characterized. Therefore it is predicted that in C9ALS/FTD patients this machinery could be either “switched off” or inefficient in the certain cell types, states or subcellular compartments thereby allowing G-quadruplex structures to form over time and exert significant pathogenic effects.

In contrast to the sense GGGGCC repeats, the antisense (CCCCGG)_n_ RNAs do not seem to form stable G-quadruplex structures; instead, hairpin structures are formed, regardless of the presence of K^+^ ions, as assessed by CD spectroscopy and native PAGE analysis (Reddy et al., 2013; Haeusler et al., 2014). Recently, more detailed solution studies of the formation of A-form-like helical conformations were conducted by using CD spectroscopy and differential scanning calorimetry (DSC; Dodd et al., 2016). The authors showed the dependence of the CCCCGG repeat number on the formation of stable helical structures: (CCCCGG)_2_, slipped intermolecular duplexes; (CCCCGG)_4_, intermolecular multimers; (CCCCGG)_10,_ intramolecular A-form like helixes. In the same study, the X-ray crystal structure of a model (CCCCGG)_3_(CCCC) RNA refined to 1.47 Å resolution was determined (Dodd et al., 2016). The identified intermolecular helix structure, likely representing the stem of the CCCCGG hairpin, had an A-form-like geometry and consisted of repeating units of four canonical Watson-Crick G-C/C-G base pairs separated by two regularly spaced tandem noncanonical C-C pairs (Figure 1E). The cytosine bases of each C-C pair were in the *anti* conformation and created one of two hydrogen bond interactions that presumably interconvert in solution: the N4 exo-amino group of one cytosine residue (H-bond donor) interacted with either the carbonyl O2 atom (N4H⋅⋅⋅O2 hydrogen bond) or with the N3 atom of the other cytosine residue (N4H⋅⋅⋅N3 hydrogen bond) (Figure 1E; Dodd et al., 2016). Moreover, perturbed electrostatic surface potential and the smaller width of the minor groove at the tandem C-C pairs (helix contraction) were observed compared to the typical A-form helix parameters. These changes are thought to be a consequence of the accommodation of the hydrogen bonding distances between the smaller size pyrimidine residues (between tandem C-C pairs).

## Cellular Proteins Abnormally Associated with the Expanded Repeat RNAs

A growing body of evidence indicates that despite affecting unrelated genes, mutant RNA-triggered toxicity is a common pathogenic mechanism involved in multiple neurodegenerative disorders associated with expanded repeats. It has been demonstrated that mutant transcripts abnormally interact with various RNA-binding proteins that deregulate many biological processes, such as alternative splicing (Osborne and Thornton, 2006), miRNA biogenesis (Sellier et al., 2013), nucleocytoplasmic transport (Tsoi et al., 2011; Sun et al., 2015; Zhang et al., 2015) or pre-rRNA processing (Tsoi et al., 2012; Haeusler et al., 2014). Over the past decade, by using more detailed analyses, the knowledge of *in vitro* RNA structures formed by expanded repeats has significantly increased. However, to gain deeper insight into the role of RNA toxicity in the pathogenesis of repeat expansion-related diseases, the interplay between mutant RNAs and their binding proteins needs to be studied in more detail. In this section, we present a few examples of repeat RNA-protein interactions for which the secondary structure formed by simple expanded repeats seems to play crucial role. Other proteins identified as RNA repeat binders are reviewed elsewhere (Jazurek et al., 2016).

### MBNL1 Interaction with Expanded CUG Repeats

The most extensively investigated simple RNA repeat-protein interaction is the one between Muscleblind-like 1 (MBNL1) protein and CUG repeat-containing RNA. It is commonly known that abnormal MBNL1 sequestration by RNA foci formed by a mutant *DMPK* transcript interferes with the proper functioning of MBNL1 in the cell, which is mainly alternative splicing regulation.

**Figure 5:**
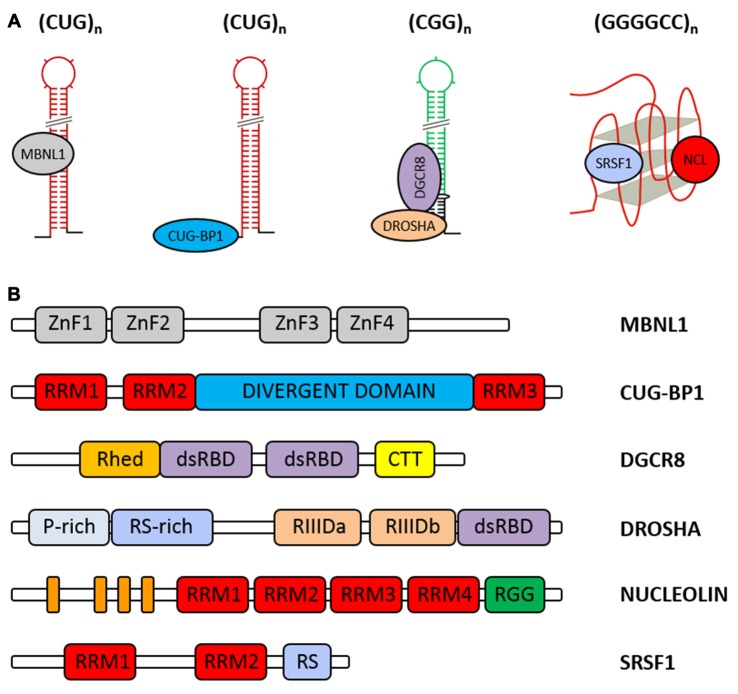
**Cellular proteins abnormally associated with the expanded repeat RNAs. (A)** Model of the selected RNA-protein interactions that occur on the expanded CUG, CGG and GGGGCC repeats. **(B)** Schematic representation of the domain architecture of human Muscleblind-like 1 (MBNL1), CUG-BP1, DGCR8, DROSHA, nucleolin (NCL) and SRSF1. RRM, RNA recognition motif; Rhed, RNA-binding heme domain; dsRBD, double-stranded RNA-binding domain; CTT, C-terminal tail; P-rich, proline rich; RS-rich, arginine/serine rich; RIIID, RNase III domain; RGG, arginine-glycine-glycine domain; the orange boxes correspond to the acidic stretches.

The human homolog of *Drosophila* MBNL1 was first identified as a protein that binds the CUG repeat in a repeat length-dependent manner (Miller et al., 2000). Experiments based on incubation of the *in vitro*-transcribed phospho-labeled *DMPK* RNAs with cellular nuclear extracts followed by UV cross-linking to capture proteins associated with these transcripts and by MS, resulted in the identification of MBNL1 as the predominantly bound protein. To verify whether the CUG repeats represent the sequence recognized by MBNL1, the investigated transcripts were limited to only CUG tracts of different lengths. It appeared that 20 and more CUG repeats cross-linked to MBNL1 with efficiency proportional to the CUG repeat length (Miller et al., 2000). Later, chemical and enzymatic structure probing of labeled RNA was employed to demonstrate that MBNL1 binds to stem portion in hairpins formed by CUG repeat tracts (Figure 5A; Yuan et al., 2007).

To determine whether MBNL1 recognizes CUG tracts *in vivo*, an yeast three-hybrid (Y3H) assay was used, which confirmed the interaction between protein and RNA through detection of the reporter gene activities (Kino et al., 2004). To confirm the three-hybrid system results, the authors also performed gel retardation assays and concluded that MBNL1 is a repeat-binding protein with a strong preference for long CUG repeat tracts. Fluorescence *in situ* hybridization (FISH) alone and in combination with IF (FISH/IF) techniques were also employed to investigate CUG repeats targeting by MBNL, which demonstrated the accumulation of long CUG tracts in nuclear foci where MBNL1 co-localized (Mankodi et al., 2001; Sznajder et al., 2016).

MBNL1 is composed of four conserved CCCH zinc-finger domains (ZnF1-4), wherein ZnF1 with ZnF2 and ZnF3 with ZnF4 are paired to form two tandems connected by a linker region (Figure 5B; Teplova and Patel, 2008; Grammatikakis et al., 2011; Konieczny et al., 2014; Sznajder et al., 2016). There are multiple MBNL1 splicing variants present in the cell, and not every isoform contains all four zinc-finger motifs. Y3H assay used for MBNL1 truncated versions showed that their ability to bind CUG triplet repeat RNA varies (Kino et al., 2004). It was postulated that only two ZnF domains and a GC dinucleotide interaction are sufficient for high-affinity MBNL1 binding to RNA (Cass et al., 2011; Purcell et al., 2012).

Electron microscopy demonstrated that MBNL1 binds to long CUG tracts as a ring-shaped structure (Yuan et al., 2007). It was postulated that MBNL1 recognizes GC-rich hairpins with pyrimidine mismatches in both physiologic and pathogenic transcripts, as previously demonstrated by others (Warf and Berglund, 2007). This finding is in agreement with previous results from Ishiura’s group, showing that MBNL1 target repeat sequences can be summarized as CHG and CHHG, where H stands for A, U or C (Kino et al., 2004). The same authors also suggested that MBNL1 prefers double-stranded RNA-containing bulges (Kino et al., 2004). Berglund’s group provided evidence that MBNL1 recognizes GC motifs embedded in pyrimidines that are present in both CUG long tracts and the natural RNA targets of the protein (Goers et al., 2010). Physiological MBNL1 targets were investigated transcriptome-wide using cross-linking and immunoprecipitation with a sequencing approach (CLIP-Seq), and as predicted, UGC- and GCU-containing four-mers were defined as MBNL1-binding sites (Wang et al., 2012).

Crystallography study revealed that MBNL1 ZnF3/4 binds to a CGCUGU single-stranded ribohexamer; thus, MBNL1 prefers single-stranded target RNAs (Teplova and Patel, 2008). To assess the structural changes in the target RNA upon MBNL1 binding, CD spectroscopy was utilized, which showed that the stability of the CUG repeat tract structure changed towards single-stranded when MBNL1 bound to the RNA (Fu et al., 2012). The authors concluded that less stable RNA structures can form more stable complexes with MBNL1. In agreement with this conclusion are results showing that MBNL1 binding is blocked when CUG repeats structure undergoes stabilization in double-stranded helical conformation by introducing pseudouridine or 2′-O-methyl modifications. Such structure stabilization inhibits MBNL1 sequestration, decreases foci size and in consequence reduces toxicity of CUG repeat containing RNA in human cells and in zebrafish (deLorimier et al., 2014). This may suggest that in contrast to *in vitro* system, RNA CUG repeats do not form double-stranded A-form structure *in vivo*.

Both the sequence and structure of the long CUG repeat RNA seem to be important for interactions with MBNL1. Although the studies mentioned above provided some insight into this interaction, the structure formed by expanded CUG repeats in RNA *in vivo* remains elusive, and many putative protein structure modulators present in the cell may play crucial roles in pathogenic transcript structure formation and in providing access to MBNL1.

### Progress in Studies on Other Repeat RNA-Protein Interactions

In addition to the MBNL1 sequestration by long CUG repeats, many other proteins that interact with neurological disease-relevant simple repeats in RNA have been identified. These proteins were mainly searched for using RNA-pulldown combined with MS or biochemical assays as reviewed in Jazurek et al. (2016). Many unique proteins belonging to various protein families and containing diverse binding domains were identified (Jazurek et al., 2016). However, research on the nature of their interaction with mutant RNAs, particularly their structural requirements, is much less advanced. In many cases, the biological consequences of such abnormal RNA-protein interactions have not been validated. Thus, there is still a need to precisely explore the mutant RNA-binding properties of these proteins. Below, we present selected examples of the most studied interactions between mutant RNA and proteins other than MBNL1.

#### CUG-BP1

CUG-BP1, which belongs to the highly conserved CUG-BP1 and ETR-3-like Factors (CELF) family of RNA-binding proteins, was one of the first proteins that was considered a factor potentially sequestered by expanded CUG repeats (Timchenko et al., 1996a,b). This protein is implicated in the control of pre-mRNA alternative splicing, mRNA stability and translation. However, further studies, including electron microscopy, revealed that CUG-BP1, in contrast to MBNL1, localizes to the single-stranded regions at the base of the hairpin structure formed by RNA containing 90 or 130 CUG repeats and 321 or 203 nt of the flanking sequence, respectively, and its binding to RNA is independent of the CUG repeat length (Michalowski et al., 1999; Mori et al., 2008; Figure 5A). As expected, CUG-BP1 did not colocalize with nuclear RNA foci in DM1 (Jiang et al., 2004; Rehman et al., 2014). More recent data obtained by NMR analysis and RNA Bind-n-Seq, which is an SELEX-based method, confirmed the binding preference of CUG-BP1 to single-stranded motifs (Edwards et al., 2013; Lambert et al., 2014). Using Y3H assay and surface plasmon resonance spectroscopy, strong associations with UG- and UGU-rich elements compared to a weak affinity for CUG repeats were observed (Takahashi et al., 2000; Kino et al., 2004; Mori et al., 2008). As demonstrated by UV crosslinking and NMR spectroscopy, the N-terminal RNA recognition motif (RRM) domains (RRM1 and RRM2) of CUG-BP1 determine the binding to CUG repeats, whereas the RRM3 domain is not involved (Figure 5B; Timchenko et al., 1999; Tsuda et al., 2009). Even though CUG-BP1 is not sequestered by the expanded CUG repeats, this protein plays a prominent role in DM1 pathogenesis. The presence of long CUG tracts within *DMPK* RNA increases the CUG-BP1 protein level, leading to aberrant splicing of multiple transcripts and, as a consequence, disease manifestation (Osborne and Thornton, 2006; Ranum and Cooper, 2006).

#### DGCR8/DROSHA

It has been demonstrated that the components of the Microprocessor complex: DROSHA nuclease and its obligate partner DGCR8, which specifically recognize and cleave pri-miRNA to pre-miRNA, also bind to the CGG repeats in a length-dependent manner (Figure 5A). DGCR8 and DROSHA were identified from pulldown analysis using long CGG stretches (60 and 100 CGG) as a bait (Sellier et al., 2013). Electrophoretic mobility shift assay (EMSA) and UV crosslinking confirmed a direct and strong interaction between DGCR8 and mutant CGG RNA but not CUG RNA (Sellier et al., 2013). This finding suggests that structural differences between hairpins formed by CGG and CUG repeats, such as the presence of U:U pairs vs. G:G base pairs, may affect DGCR8 binding to mutant RNA. Both proteins colocalize within CGG RNA aggregates (Sellier et al., 2013). Although the CGG binding properties of DGCR8 and DROSHA have been demonstrated, the detailed structural requirements for CGG recognition are still unknown. Both proteins belong to double-stranded RNA-binding proteins that contain double-stranded RNA-binding domains (dsRBDs) consisting of a *α-β-β-β-α* fold that recognizes A-form dsRNA, which is believed to also be formed by CGG repeats (structural studies; Saunders and Barber, 2003; Tian et al., 2004; Masliah et al., 2013; Figure 5B). DROSHA has a conserved central domain that is essential for its cleavage activity, two RNase III domains (RIIID) and one dsRBD. However, because of the weak RNA-binding capacity of dsRBD, to recognize pri-miRNA, DROSHA needs a DGCR8 (Denli et al., 2004; Gregory et al., 2004; Han et al., 2004, 2006). Based on recent data, dimerized dsRBD and RNA-binding heme domains (Rhed) of DGCR8 interact with the upper stem and apical loop of bound pri-miRNA, whereas DROSHA not only serves as the catalytic subunit but also determines the cleavage sites by recognizing the basal junction of pri-miRNA and measuring the length of dsRNA from the basal junction (Nguyen et al., 2015; Kwon et al., 2016). Is it possible that similar RNA-protein interactions could exist in the case of CGG repeats and DGCR8-DROSHA? It has been reported that DGCR8 binds to expanded repeats and pri-miRNAs such as pri-miR-124, pri-miR-125 and pri-Let-7 with similar affinity. However, the DGCR8-DROSHA interaction with long CGG repeats does not result in cleavage by DROSHA of mutant RNA into shorter CGG hairpins. It is likely that differences between the structures of pri-miRNA and CGG repeats affect the activity of DROSHA (Sellier et al., 2013). Moreover, it has been reported that G residues rarely occur within DROSHA cleavage sites (Starega-Roslan et al., 2015). It is worth noting that small compounds that tightly bind to RNAs containing repeated non-Watson-Crick GG pairs such as the ones that are present in CGG repeats inhibit the interaction between DGCR8 and (CGG)_12_ (Disney et al., 2012; Tran et al., 2014; Yang et al., 2016). The consequence of DGCR8-DROSHA titration by long CGG repeat hairpins is reduced processing of pri-miRNAs, which might lead to neuronal dysfunction and cell death (Sellier et al., 2013).

#### NUCLEOLIN

One of the established binders of GGGGCC G-quadruplexes is nucleolin (NCL), a multifunctional protein involved in DNA metabolism, transcription, ribosome assembly, mRNA stability and translation (Almeida et al., 2013; Haeusler et al., 2014). Previous studies revealed that NCL interacts with DNA and RNA quadruplexes, leading to, e.g., the stabilization of the G-quadruplex structure (Brázda et al., 2014). The properties of NCL binding to the GGGGCC repeats were identified for the first time in RNA pulldown assays followed by MS using either biotinylated (GGGGCC)_4_ or (GGGGCC)_30_ (Almeida et al., 2013; Haeusler et al., 2014; Figure 5A). The interaction of NCL with mutant RNA is structure dependent, as NCL binds only GGGGCC repeats in the G-quadruplex structure (Haeusler et al., 2014; Cooper-Knock et al., 2015). Such an association was not observed for (GGGGCC)_4_ when conditions favoring hairpin structure formation were used (without K^+^ ions) or when antisense hairpin-forming (CCCCGG)_4_ repeats were studied. NCL directly binds to the G-quadruplex motif, as demonstrated by RNA pulldown with GST-NCL, and colocalizes with GGGGCC RNA foci. It is still unknown which domain of NCL is involved in the interaction with GGGGCC G-quadruplexes. However, based on relevant data from c-*MYC* G-quadruplex structures, it is likely that the GGGGCC G-quadruplex-binding domain might consist of RRM3, RRM4 and the arginine-glycine-glycine domain (RGG; González and Hurley, 2010; Figure 5B). The consequence of NCL sequestration is impaired function of the nucleolus (Haeusler et al., 2014). It should be mentioned that NCL also interacts with the expanded CAG repeats (Tsoi et al., 2012). Importantly, when CAG continuity was disrupted by the presence of the CAA triplet, interaction with NCL was not observed. RRM2 and RRM3 domains of NCL determine NCL binding to the mutant RNA. As described for GGGGCC repeats, abnormal interaction between NCL and the expanded CAG repeats also results in the induction of nuclear stress (Tsoi et al., 2012).

#### SRSF1

The splicing factor SRSF1 (also known as SF2/ASF), a member of the arginine/serine-rich splicing factor protein family, which is involved in constitutive and alternative splicing, mRNA export and decay, or translation, represents another GGGGCC quadruplex-binding protein (Figures 5A,B). The GGGGCC quadruplex binding properties of SRSF1 were determined by EMSA using radiolabeled (GGGGCC)_4_ and (GGGGCC)_8_ (Reddy et al., 2013; Zamiri et al., 2014). RNA-protein complexes were not observed when antisense hairpin forming (CCCCGG)_4_ was used in the analysis (Reddy et al., 2013). Moreover, SRSF1 interaction with (GGGGCC)_8_ was abolished in the presence of cationic porphyrin TMPyP4, a known RNA G-quadruplex destabilizer (Zamiri et al., 2014). Additionally, SRSF1 is trapped by nuclear aggregates formed by GGGGCC repeats (Rossi et al., 2015). It is still unresolved how SRSF1 sequestration is implicated in the pathogenesis of C9ALS/FTD.

#### hnRNPs

Some of the hnRNPs—RNA binding proteins which regulate pre-mRNA processing and other aspects of mRNA metabolism and transport, exhibit preference for binding to GGGGCC-quadruplexes. One of these proteins is hnRNP U, whose interaction with mutant RNA strictly depends on the formation of the G-quadruplex structure (Haeusler et al., 2014). Some data suggests that the G-quartet structure-dependent binding may also occur for hnRNP A1 and hnRNP H. As revealed by EMSA, TMPyP4 disturbs interaction of hnRNP A1 with (GGGGCC)_8_ (Zamiri et al., 2014). Using a UV-crosslinking assay it was demonstrated that G-quadruplexes formed by the (GGGGCC)_10_ are mainly associated with hnRNP H (Conlon et al., 2016). The observed interaction was reduced in the presence of G-quadruplex destabilizing GTP analog. Additionally, hnRNP H colocalizes with GGGGCC quadruplex aggregates as shown by using antibody specifically recognizing G-quadruplex structure. However, based on the results of EMSA analysis which used RNA that either formed or not G-quartet structure, hnRNP H might have binding preference to RNAs with linear G-tracts. Biological consequence of hnRNP H sequestration is dysregulated splicing of multiple hnRNP H target transcripts in patients with C9ALS (Conlon et al., 2016). As hnRNP A1 and hnRNP H can also bind antisense CCCCGG repeats and in case of hnRNP H also CUG repeats, it is likely that these proteins recognize both G-quadruplex and hairpin structures (Kim et al., 2005; Cooper-Knock et al., 2015).

## Conclusions and Further Directions

Despite considerable progress in the structural determination of disease-relevant RNAs with the expanded repeats, many questions regarding the pathomechanisms of repeat expansion disorders remain unanswered. The data obtained during the last 20 years clearly show that precise deciphering of the disease-triggering mechanisms by focusing only on the RNA structure is not possible. It is a well-supported view that abnormal structure-dependent interactions of expanded RNA repeats with various cellular proteins might be the main or contributing factor of repeat expansion disorders. Based on the RNA-protein sequestration model, mutant transcripts that form nuclear aggregates are potent traps for RNA-binding proteins. Such an association results mostly in the loss of function of these proteins, which leads to the deregulation of many important cellular processes. Therefore, to understand the ambiguous pathomechanisms of repeat expansion diseases triggered by RNA, it is crucial to establish: (1) the exact structure of full-length repeat-containing transcripts in cells; (2) whether there are any alternative RNA structures that are formed in cells by the same repeat-containing transcripts; (3) the complement of proteins that bind to the specific RNA repeats; (4) the exact nature of these RNA-protein interactions; (5) which of these RNA-protein interactions exhibit toxic effects to cells; and (6) how this toxic effect is manifested (which cellular processes are affected).

To date, the structural studies on simple repeat-containing transcripts were performed mostly under non-physiological conditions and relied on a range of biophysical methods, such as X-ray crystallography and NMR and CD spectroscopies or on traditional enzymatic (S1, Mung Bean nuclease and RNase A, T1, T2 and V1) and chemical (Pb^2+^ ions) RNA structure probing. These biophysical studies shed light on the physical characteristics of the RNA structures formed by the repeated sequences, i.e., thermodynamic stabilities, detailed duplex/hairpin properties and hydrogen bonding status; however, these studies were performed only on isolated, short repeat-containing oligomers without native sequence context. Such studies were able to provide very detailed, but only partial, information on the structures formed by the full-length transcripts in cells. This finding also applies to the enzymatic and chemical RNA structure probing experiments in which relatively short 5′ and 3′ repeat-flanking sequences were analyzed, which are rather insufficient, in light of recent findings showing that extensive long-range intra- and intermolecular RNA-RNA interactions occur in cells (Aw et al., 2016; Lu et al., 2016; Sharma et al., 2016). Therefore, there is a need to perform detailed analysis of the *in vivo* formed full-length repeat-containing transcripts using recently developed, cutting-edge RNA structural approaches that take advantage of high-throughput, next-generation sequencing (NGS) technologies. Currently, the focus should be to harness these *in vivo* RNA structural probing methods or to develop novel ones to solve the precise RNA structures of repeat-containing transcripts with single-nucleotide resolution.

Due to technical challenges, the majority of the studies that focused on identifying proteins that bind to repeat-containing transcripts and probing in detail the structural properties of these RNA-protein interactions were performed by using traditional *in vitro* strategies. The approaches used included RNA pull-down followed by either MS analysis (an unbiased comprehensive approach) or immunoblotting against proteins predicted to interact with repeat-containing transcripts (a biased “candidate” protein approach), EMSA, filter binding assay, *in vitro* RNA immunoprecipitation, UV-induced crosslinking and RNase footprinting assays, fluorescence polarization anisotropy and electron microscopy studies (Jazurek et al., 2016). As the above strategies rely on *in vitro*-transcribed repeat-containing transcripts that can potentially adopt nonphysiological RNA structures that do not match those inside the cells, the analysis of RNA-protein interactions might produce both false-positive and false-negative results.

Regarding inherited disadvantages of the previously used approaches to identify proteins that associate with repeat-containing transcripts, there is currently a need to streamline recently developed, high-throughput, cutting-edge methods or to develop novel assays in order to identify novel proteins that are stably or transiently trapped/sequestered by expanded repeat-containing transcripts in RNA foci. This goal can potentially be achieved with the use of unbiased *in vivo* RNA pull-down strategies relying on either CRISPR/RdCas9 (O’Connell et al., 2014; Nelles et al., 2016) or CRISPR/C2c2 (Abudayyeh et al., 2016) technologies or crosslinking-MS analysis (Schmidt et al., 2012; Kramer et al., 2014). In the case of newly developed CRISPR-based technologies, purification of endogenously expressed unmodified RNAs with increased specificity, together with interacting protein partners, followed by MS identification can be accomplished. Moreover, high-throughput quantitative proteomics strategies (Tsai et al., 2011; Klass et al., 2013; Chen et al., 2015) could be harnessed to investigate both spatial and temporal repeat-binding proteomes, which are thought to be dynamic and to depend on cell cycle progression and changes during different stages of the RNA life cycle. Last, in order to determine the exact protein-binding sites within repeat-containing transcripts, high-throughput technologies that exploit improved *in vivo* RIP analysis followed by NGS (CLIP-based methods) can be of use, i.e., CLIP (Ule et al., 2005; Jensen and Darnell, 2008), HiTS-CLIP (Licatalosi et al., 2008; Zhang and Darnell, 2011), PAR-CLIP (Hafner et al., 2010; Ascano et al., 2012), iCLIP (König et al., 2010; Huppertz et al., 2014) and eCLIP (Conway et al., 2016; Van Nostrand et al., 2016).

Novel findings regarding the RNA structures formed *in vivo* by repeat-containing transcripts as well as detailed interaction analysis of newly identified proteins that are sequestrated by these toxic RNAs will not only enable the researchers to better understand the RNA toxicity in repeat expansion-related disorders but will also provide rational design principles for RNA structure-based therapies to combat these diseases.

## Author Contributions

AC, MJ, KD and WJK wrote and revised this review article.

## Funding

This work was supported by a grant from the National Science Centre (2012/06/A/NZ1/00094 to WJK, 2014/15/B/NZ1/01880 to WJK, 2015/19/B/NZ2/02453 to WJK, 2015/19/D/NZ5/02183 to MJ, 2015/16/S/NZ1/00086 to KD) and by the Polish Ministry of Science and Higher Education, under the KNOW program for years 2014–2019.

## Conflict of Interest Statement

The authors declare that the research was conducted in the absence of any commercial or financial relationships that could be construed as a potential conflict of interest.
